# Crystal structure of defect scheelite-type Nd_2/3_[WO_4_]

**DOI:** 10.1107/S2414314624001755

**Published:** 2024-03-06

**Authors:** Benjamin Knies, Ingo Hartenbach

**Affiliations:** a University of Stuttgart, Institute of Inorganic Chemistry, Pfaffenwaldring 55, 70569 Stuttgart, Germany; Vienna University of Technology, Austria

**Keywords:** crystal structure, oxidotungstate(VI), neodymium, scheelite

## Abstract

Neodymium(III) *ortho*-oxidotungstate(VI) was synthesized as a side-product in an unsuccessful synthesis attempt at fluoride derivatives of neodymium tungstate in fused silica ampoules, using neodymium(III) oxide, neodymium(III) fluoride and tungsten trioxide. Violet, platelet-shaped single crystals of the title compound emerged of the bulk, which crystallize in the defect scheelite type with a trigonal dodeca­hedral coordination of oxide anions around the Nd^3+^ cations and the hexa­valent tungsten cations situated in the centers of oxide tetra­hedra.

## Abstract 2

The crystal structure of defect-scheelite type Nd_2/3_[WO_4_] consists of [WO_4_]^2−^ tetra­hedra and trigonal [NdO_8_]^13–^ dodeca­hedra.

## Graphical abstract

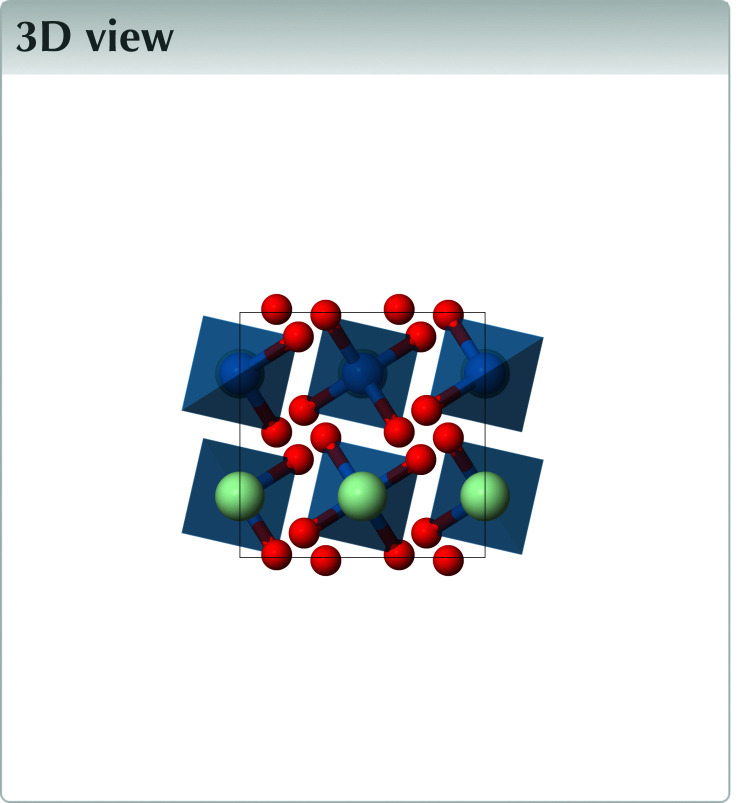

## Structure description

**Figure 1:**
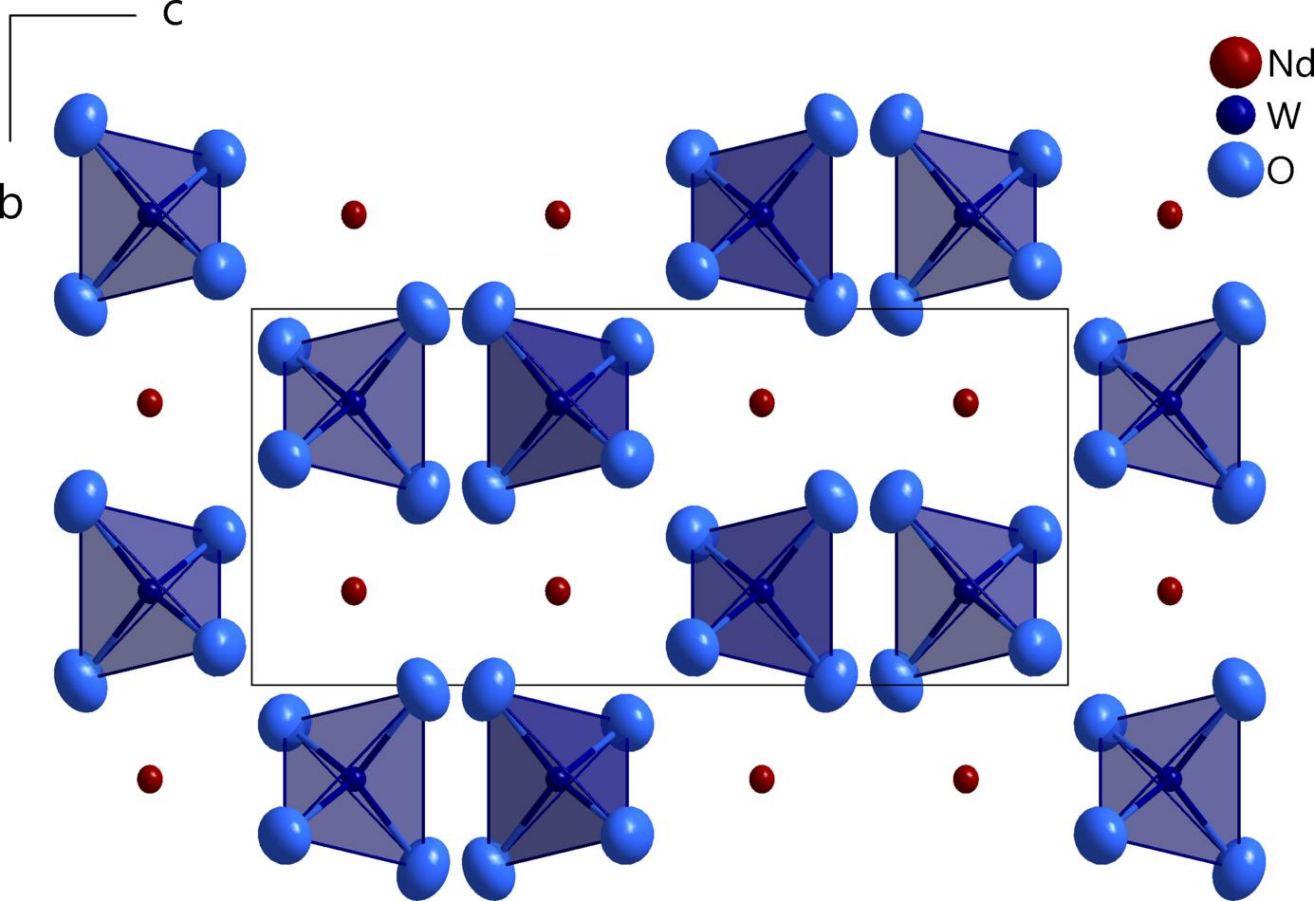
Augmented unit cell of Nd_2/3_[WO_4_] in a view along [100], with [WO_4_]^2−^ anions in polyhedral representation and displacement ellipsoids drawn at the 95% probability level.

**Figure 2:**
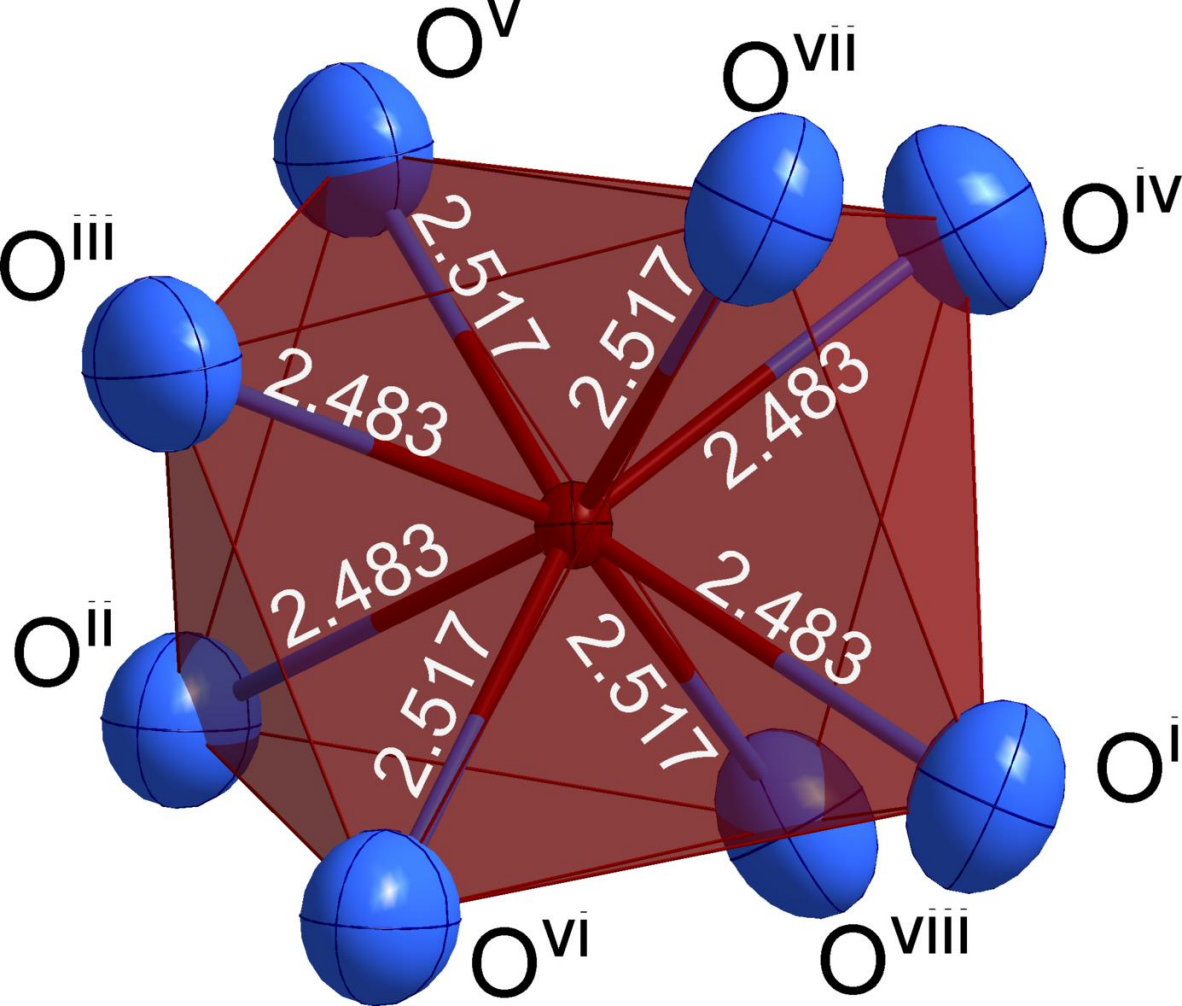
Cationic coordination sphere around the Nd^3+^ cation in the shape of a trigonal [NdO_8_]^13−^ dodeca­hedron; displacement ellipsoids are drawn at the 95% probability level. [Symmetry codes: (i) *y* − 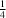 , −*x* + 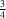 , *z* + 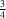 ; (ii) *x* − 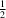 , *y*, −*z* + 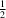 ; (iii) −*x* + 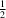 , −*y* + 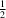 , −*z* + 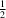 ; (iv) −*y* + 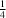 , *x* − 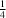 , *z* + 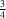 ; (v) *x* − 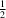 , *y* − 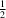 , *z* + 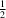 ; (vi) −*x* + 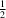 , −*y* + 1, *z* + 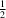 ; (vii) −*y* + 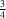 , *x* − 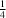 , −*z* + 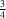 ; (viii) *y* − 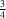 , −*x* + 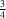 , −*z* + 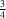 ].

Nd_2/3_[WO_4_] crystallizes in the defect scheelite structure type (space group *I*4_1_/*a*, Dickinson, 1920 ▸; see Fig. 1 ▸). The tungsten cations (Wyckoff position 4*a*, site symmetry 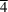 ) form regular tetra­hedra [WO_4_]^2−^ together with four oxide anions, exhibiting a bond length of 1.783 (4) Å. The neodymium cations (Wyckoff position 4*b*, site symmetry 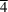 ) are coord­inated by eight oxide anions, forming a slightly distorted trigonal dodeca­hedron, in which two different bond lengths, 2.483 (4) Å and 2.516 (4) Å, each one appearing four times, are found (Fig. 2 ▸). The distance between two neodymium cations is 3.9116 (2) Å. The corresponding oxidomolybdate(IV), Nd_2/3_[MoO_4_], crystallizes in the same structure type (Schustereit *et al.*, 2011 ▸).

Another, formula-analogous polymorph of neodymium(III) *ortho*-oxidotungstate(VI) with the composition Nd_2_[WO_4_]_3_ is already known in literature (Weil *et al.*, 2009 ▸), with this compound crystallizing in the *scheelite*-derived Eu_2_[WO_4_]_3_ structure type (space group *C*2/*c*; Templeton & Zalkin, 1963 ▸). The main difference between these polymorphs is the emergence of a fifth, slightly longer distance from W^6+^ to O^2−^ in Nd_2_[WO_4_]_3_, resulting in [W_2_O_8_]^4–^ entities, built of two edge-sharing rectangular pyramids being present in its crystal structure, while in the title compound Nd_2/3_[WO_4_] the [WO_4_]^2−^ tetra­hedra remain isolated from each other. A rare-earth metal oxidotungstate(VI), crystallizing in the scheelite*-*type with a fully occupied cationic position is known with Eu^2+^ cations, namely Eu[WO_4_] (López-Moreno *et al.*, 2011 ▸).

## Synthesis and crystallization

Single-crystals of Nd_2/3_[WO_4_] formed in an unsuccessful synthesis attempt to achieve fluoride derivatives of neodym­ium tungstate, which was performed in fused silica ampoules, utilizing neodymium trifluoride, neodymium(III) oxide and tungsten trioxide as starting materials at approximately 1123 K. The crystals emerged as violet platelets and remained stable under atmospheric conditions.

## Refinement

Crystal data, data collection and structure refinement details are summarized in Table 1 ▸. The site occupancy of the neo­dym­ium cations was fixed at 2/3 to maintain electroneutrality.

## Supplementary Material

Crystal structure: contains datablock(s) I. DOI: 10.1107/S2414314624001755/wm4207sup1.cif

Structure factors: contains datablock(s) I. DOI: 10.1107/S2414314624001755/wm4207Isup2.hkl

CCDC reference: 2312842

Additional supporting information:  crystallographic information; 3D view; checkCIF report

## Figures and Tables

**Table 1:** Experimental details

Crystal data	
Chemical formula	Nd_2/3_[WO_4_]
*M*_r_	344.01
Crystal system, space group	Tetragonal, *I*4_1_/*a*
Temperature (K)	293
*a*, *c* (Å)	5.3048 (3), 11.4999 (9)
*V* (Å^3^)	323.62 (4)
*Z*	4
Radiation type	Mo *K*α
μ (mm^−1^)	45.98
Crystal size (mm)	0.09 × 0.08 × 0.06

Data collection	
Diffractometer	Stoe *IPDS*
Absorption correction	Numerical [*X-SHAPE* (Stoe & Cie, 1997 ▸); *HABITUS* (Herrendorf, 1995 ▸)]
*T*_min_, *T*_max_	0.015, 0.060
No. of measured, independent and observed [*I* > 2σ(*I*)] reflections	2201, 291, 231
*R*_int_	0.061
(sin θ/λ)_max_ (Å^−1^)	0.754

Refinement	
*R*[*F* ^2^ > 2σ(*F* ^2^)], *wR*(*F* ^2^), *S*	0.022, 0.050, 1.01
No. of reflections	291
No. of parameters	15
Δρ_max_, Δρ_min_ (e Å^−3^)	1.08, −1.26

Computer programs: *DIF4* and *REDU4* (Stoe & Cie, 1991 ▸), *SHELXT* (Sheldrick, 2015*a* ▸), *SHELXL* (Sheldrick, 2015*b* ▸), *DIAMOND* (Brandenburg & Putz, 2005 ▸) and *publCIF* (Westrip, 2010 ▸).

## full crystallographic data

### Crystal data

**Table:**

Nd_0.67_[WO_4_]	*D*_x_ = 7.061 Mg m^−^^3^
*M**_r_* = 344.01	Mo *K*α radiation, λ = 0.71073 Å
Tetragonal, *I*4_1_/*a*	Cell parameters from 1393 reflections
*a* = 5.3048 (3) Å	θ = 2.9–33.0°
*c* = 11.4999 (9) Å	µ = 45.98 mm^−^^1^
*V* = 323.62 (4) Å^3^	*T* = 293 K
*Z* = 4	Platelet, violet
*F*(000) = 584	0.09 × 0.08 × 0.06 mm

### Data collection

**Table:**

Stoe IPDS diffractometer	291 independent reflections
Radiation source: fine-focus sealed tube	231 reflections with *I* > 2σ(*I*)
Graphite monochromator	*R*_int_ = 0.061
ω–scans	θ_max_ = 32.4°, θ_min_ = 4.2°
Absorption correction: numerical [X-SHAPE (Stoe & Cie, 1997); HABITUS (Herrendorf, 1995)]	*h* = −8→7
*T*_min_ = 0.015, *T*_max_ = 0.060	*k* = −8→7
2201 measured reflections	*l* = −17→17

### Refinement

**Table:**

Refinement on *F*^2^	0 restraints
Least-squares matrix: full	*w* = 1/[σ^2^(*F*_o_^2^) + (0.0265*P*)^2^] where *P* = (*F*_o_^2^ + 2*F*_c_^2^)/3
*R*[*F*^2^ > 2σ(*F*^2^)] = 0.022	(Δ/σ)_max_ < 0.001
*wR*(*F*^2^) = 0.050	Δρ_max_ = 1.08 e Å^−^^3^
*S* = 1.01	Δρ_min_ = −1.26 e Å^−^^3^
291 reflections	Extinction correction: SHELXL (Sheldrick, 2015b), Fc^*^=kFc[1+0.001xFc^2^λ^3^/sin(2θ)]^-1/4^
15 parameters	Extinction coefficient: 0.088 (4)

### Special details

**Table:**

Geometry. All esds (except the esd in the dihedral angle between two l.s. planes) are estimated using the full covariance matrix. The cell esds are taken into account individually in the estimation of esds in distances, angles and torsion angles; correlations between esds in cell parameters are only used when they are defined by crystal symmetry. An approximate (isotropic) treatment of cell esds is used for estimating esds involving l.s. planes.

### Fractional atomic coordinates and isotropic or equivalent isotropic displacement parameters (Å^2^)

**Table:**

	*x*	*y*	*z*	*U*_iso_*/*U*_eq_	Occ. (<1)
Nd	0.000000	0.250000	0.625000	0.0163 (2)	0.6667
W	0.000000	0.250000	0.125000	0.0135 (2)	
O	0.2382 (8)	0.3998 (7)	0.0401 (3)	0.0221 (8)	

### Atomic displacement parameters (Å^2^)

**Table:**

	*U*^11^	*U*^22^	*U*^33^	*U*^12^	*U*^13^	*U*^23^
Nd	0.0176 (3)	0.0176 (3)	0.0139 (4)	0.000	0.000	0.000
W	0.0138 (2)	0.0138 (2)	0.0130 (3)	0.000	0.000	0.000
O	0.026 (2)	0.022 (2)	0.0185 (17)	0.0001 (15)	0.0047 (16)	0.0011 (16)

### Geometric parameters (Å, º)

**Table:**

Nd—O^i^	2.483 (4)	Nd—Nd^ix^	3.9116 (2)
Nd—O^ii^	2.483 (4)	Nd—Nd^x^	3.9116 (2)
Nd—O^iii^	2.483 (4)	Nd—Nd^xi^	3.9116 (2)
Nd—O^iv^	2.483 (4)	Nd—Nd^xii^	3.9116 (2)
Nd—O^v^	2.516 (4)	W—O^xiii^	1.783 (4)
Nd—O^vi^	2.516 (4)	W—O^xiv^	1.783 (4)
Nd—O^vii^	2.516 (4)	W—O^xv^	1.783 (4)
Nd—O^viii^	2.516 (4)	W—O	1.783 (4)
			
O^i^—Nd—O^ii^	125.78 (12)	O^v^—Nd—O^vii^	98.65 (6)
O^i^—Nd—O^iii^	125.78 (12)	O^vi^—Nd—O^vii^	98.65 (7)
O^ii^—Nd—O^iii^	80.25 (19)	O^i^—Nd—O^viii^	68.34 (10)
O^i^—Nd—O^iv^	80.2 (2)	O^ii^—Nd—O^viii^	72.96 (8)
O^ii^—Nd—O^iv^	125.78 (12)	O^iii^—Nd—O^viii^	152.39 (16)
O^iii^—Nd—O^iv^	125.78 (12)	O^iv^—Nd—O^viii^	77.05 (15)
O^i^—Nd—O^v^	152.39 (16)	O^v^—Nd—O^viii^	98.65 (7)
O^ii^—Nd—O^v^	68.34 (10)	O^vi^—Nd—O^viii^	98.65 (6)
O^iii^—Nd—O^v^	77.05 (15)	O^vii^—Nd—O^viii^	134.35 (18)
O^iv^—Nd—O^v^	72.96 (8)	O^xiii^—W—O^xiv^	107.43 (13)
O^i^—Nd—O^vi^	72.96 (8)	O^xiii^—W—O^xv^	107.43 (13)
O^ii^—Nd—O^vi^	77.05 (15)	O^xiv^—W—O^xv^	113.6 (3)
O^iii^—Nd—O^vi^	68.34 (10)	O^xiii^—W—O	113.6 (3)
O^iv^—Nd—O^vi^	152.39 (16)	O^xiv^—W—O	107.43 (13)
O^v^—Nd—O^vi^	134.35 (18)	O^xv^—W—O	107.43 (13)
O^i^—Nd—O^vii^	77.05 (15)	W—O—Nd^iii^	132.2 (2)
O^ii^—Nd—O^vii^	152.39 (16)	W—O—Nd^xvi^	120.51 (19)
O^iii^—Nd—O^vii^	72.96 (8)	Nd^iii^—O—Nd^xvi^	102.95 (15)
O^iv^—Nd—O^vii^	68.34 (10)		

Symmetry codes: (i) *y*−1/4, −*x*+3/4, *z*+3/4; (ii) *x*−1/2, *y*, −*z*+1/2; (iii) −*x*+1/2, −*y*+1/2, −*z*+1/2; (iv) −*y*+1/4, *x*−1/4, *z*+3/4; (v) *x*−1/2, *y*−1/2, *z*+1/2; (vi) −*x*+1/2, −*y*+1, *z*+1/2; (vii) −*y*+3/4, *x*−1/4, −*z*+3/4; (viii) *y*−3/4, −*x*+3/4, −*z*+3/4; (ix) −*x*, −*y*, −*z*+1; (x) −*x*+1/2, −*y*+1/2, −*z*+3/2; (xi) −*x*−1/2, −*y*+1/2, −*z*+3/2; (xii) −*x*, −*y*+1, −*z*+1; (xiii) −*x*, −*y*+1/2, *z*; (xiv) −*y*+1/4, *x*+1/4, −*z*+1/4; (xv) *y*−1/4, −*x*+1/4, −*z*+1/4; (xvi) *x*+1/2, *y*+1/2, *z*−1/2.
